# Survey of infection control resources and services in outpatient settings in China

**DOI:** 10.1186/2047-2994-4-S1-P249

**Published:** 2015-06-16

**Authors:** F Qiao, W Huang, Y Wang, Z Zong

**Affiliations:** 1 Infection Control Department, West China Hospital, Sichuan University, Chengdu, China

## Objectives

This study was conducted to assess the status of infection control resources and services in outpatient settings in China.

## Methods

A questionnaire on the structure, organization, and resources for infection surveillance and control work was designed and sent to 420 hospitals distributed in 5 provinces in China. The questionnaire also included the jobs that have carried out in these hospitals. Epidata 3.1 and SPSS 17.0 were used for data entry and analyze.

## Results

A total of 416 (99.0%) hospitals responded, including 115 (27.6%) tertiary hospitals, 131 (31.5%) second-class hospitals, 39 (9.4%) traditional Chinese Medicine hospitals, 38 (9.1%) women’s and children’s hospitals, 30 (7.2%) private hospitals, 50 (12.0%) community health service centers and 13 (3.2%) other type hospitals. 94.7% of the participating hospitals have set up an infection control team in outpatient settings, 94.5% of them had an surveillance and control program, but only 75.5% outpatient settings arranged an link nurse to do infection control job. In terms of hand hygiene, 372 (89.4%) hospitals had hand sinks and 343 (82.5%) provided alcohol based hand rub in every clinic rooms. Only 76 (18.3%) outpatient settings offered hand hygiene equipment for patients in the waiting room. For personal protective equipment, 290 (69.7%) hospitals provided gloves for health care workers (HCWs), and only 270 (64.9%) and 181 (43.5%) settings afforded surgical mask and respirator for health care workers respectively. 400 (96.2%) hospitals gave HCWs education or training on infection control. Observe hand hygiene compliance (348, 83.7%) and supervise disinfection of health care surroundings (330, 79.3%) were the main infection control activity carried out in these settings, only a few settings did another infection work such as surveillance for surgical site infection (9.6%) or catheter related bloodstream infection (9.1%).

## Conclusion

As a developing country, resources for infection prevention and control in outpatient settings were not enough in China. Only a few hospitals did health care associated infection surveillance and control work besides education. More needs to be done to improve the current situation of infection prevention and control in China.

## Disclosure of interest

None declared.

